# Acceptability of a Dolutegravir Oral Dispersible Film in Term Neonates Born to Mothers Living With HIV

**DOI:** 10.1002/jia2.70104

**Published:** 2026-06-19

**Authors:** Lario Viljoen, Charlene Purdy, Verah Luke, Samantha du Toit, Marisa Groenewald, Lindee Ganger, Anneke C. Hesseling, Anthony J. Garcia‐Prats, Tim R. Cressey, Adrie Bekker

**Affiliations:** ^1^ Desmond Tutu TB Centre, Department of Paediatrics and Child Health, Faculty of Medicine and Health Sciences Stellenbosch University Cape Town South Africa; ^2^ Family Centre for Research with Ubuntu (FAMCRU), Department of Paediatrics and Child Health, Faculty of Medicine and Health Sciences Stellenbosch University Cape Town South Africa; ^3^ Department of Pediatrics, School of Medicine and Public Health University of Wisconsin‐Madison Madison Wisconsin USA; ^4^ AMS‐PHPT Research Collaboration, Department of Medical Technology, Faculty of Associated Medical Sciences Chiang Mai University Chiang Mai Thailand

**Keywords:** HIV prevention, HIV treatment, infants, novel formulations, oral film, qualitative

## Abstract

**Introduction:**

Dolutegravir (DTG) is a key antiretroviral (ARV) drug for children living with HIV, but no specific formulation is available for neonates (<28 days old). One of the DTG formulations evaluated in the PETITE‐DTG trial was a novel 5 mg oral DTG dispersible film (DTG‐Film). Acceptability to end‐users is essential when introducing new ARV drugs. We report on the experiences of mothers and health workers related to the DTG‐Film.

**Methods:**

PETITE‐DTG was a phase I/II, open‐label two‐stage study evaluating the pharmacokinetics, safety and acceptability of two paediatric DTG formulations in term neonates in South Africa. In the multi‐dose stage, 43 term neonates born to women with HIV were randomized to receive either the 5 mg DTG‐Film or half of a 10 mg DTG dispersible tablet, in addition to zidovudine syrup prophylaxis, for 28 days. In‐depth interviews were conducted in a sub‐set of mothers whose neonates received DTG‐Film (at three time points) and with health workers (at two time points). Thematic analysis was employed.

**Results:**

Data were collected (September 2023–October 2024) from 16 virologically suppressed mothers (median age: 38 years) whose neonates received DTG‐Film and six female health workers (median age: 47 years) involved in the study. Usability was described positively—with participants highlighting ease of administration, quick dissolution, accurate dosing with no spillage and convenient packaging. Some mothers were initially hesitant due to unfamiliarity with the film, but after 1–2 doses, most reported liking or preferring the DTG‐Film above other known ARV formulations. The film integrated well into households, was supported by family members and was considered discreet, helping to avoid unintentional HIV‐status disclosure. Previous experience with neonatal ARV drugs and trust in health workers supported acceptability. Health workers noted DTG‐Film was potentially fit for use in public health settings, where other formulations require manipulation prior to administration. Health workers played a key role in reassuring mothers that the “paper” was indeed medicine.

**Conclusions:**

Mothers and health workers found the new DTG‐Film acceptable for neonatal use. While initial hesitancy was noted, acceptability increased with use. Targeted peer support and engagement with mothers and health workers will be essential to familiarize end‐users with the novel DTG‐Film.

## 1 Introduction

Globally, there are more than 39 million people living with HIV, including 20 million women of reproductive age (15 years old and above). It is estimated that 1.3 million women with HIV are pregnant each year, with the vast majority residing in sub‐Saharan Africa [1]. All of their newborns require antiretroviral (ARV) formulations for prevention or treatment of HIV. Between 2010 and 2024, global vertical‐transmission‐prevention (VTP) programmes achieved 62% reduction in HIV acquisitions in children under 5 [2]. However, remaining HIV transmissions still occur at birth and during the breastfeeding period [1, 3]. Elimination of vertical HIV transmission in neonates (<28 days of life) will not be achieved without the equitable access of more acceptable and potent neonatal ARV formulations [4].

Current available neonatal ARV formulations are mostly in syrup form and from older, more toxic ARV drug classes [5]. Dolutegravir (DTG)‐based ARV treatment is the preferred WHO first‐ and second‐line regimen for adults and children, but no tailored DTG formulation is available for neonates [6]. Until recently, DTG dosing guidance was available only for infants aged  ≥4 weeks (weighing ≥3 kg), thereby excluding neonates from equitable access to optimal ARVs [7]. For the WHO weight band of 3 to <6 kg, the recommended DTG dose is 5 mg once daily. The PETITE‐DTG trial (NCT05590325) was designed to evaluate the pharmacokinetics, safety and acceptability of two paediatric DTG formulations in neonates; a novel 5 mg oral DTG dispersible film (DTG‐Film), and half of a generic scored 10 mg dispersible tablet (DTG‐DT). These 5 mg DTG formulations, developed for children, were adapted for use in term neonates, applying an innovative dosing strategy of 5 mg DTG every second day for the first 2 weeks of life, followed by 5 mg DTG every day through 4 weeks of life. DTG was administered in addition to zidovudine (ZDV) syrup to ensure optimal HIV prophylaxis for these neonates at low risk of HIV acquisition [8, 9].

As a first of its kind, the PETITE‐DTG trial evaluated a 5 mg oral dispersible film (DTG‐Film). Recent developments in oral drug delivery systems include oral dispersible films, consisting mainly of single‐ or multilayer thin polymer‐based sheets, usually placed in the mouth where they disperse rapidly. These films are developed to disintegrate in saliva while the clinical effect of the drug is enhanced via pre‐gastric absorption in the saliva [10]. Studies have reported that these films are easy to administer and often preferred above existing formulations, including syrups, providing alternative options when care is provided for neonates [11, 12]. However, oral dispersible films are not widely available and, prior to the PETITE‐DTG trial, have not yet been formulated for ARV drugs. Here, we describe the acceptability of the DTG‐Film among end‐users (mothers and health workers) during the first month of life in term neonates born to mothers living with HIV.

## 2 Methods

### 2.1 Design and Setting

The PETITE‐DTG trial was a phase I/II, open‐label, single‐arm, two‐stage pharmacokinetic, safety and acceptability trial. This acceptability assessment was a nested longitudinal qualitative assessment of treatment acceptability among mothers with neonates enrolled in the trial and health workers familiar with the trial.

This study was implemented at the Family Centre for Research with Ubuntu (FAMCRU), a clinical research centre at Tygerberg Hospital, Cape Town, South Africa. Tygerberg Hospital is a large secondary and tertiary referral centre for ∼8000 annual high‐risk pregnancies in the Western Cape Province [13]. It serves a diverse population of ∼2.6 million people from the surrounding communities [13]. The antenatal HIV prevalence in the province is estimated to be 16.3% based on the most recent national survey [14]. Patients mostly speak English, Afrikaans or isiXhosa. Health workers are fluent in English and frequently one other local language.

Current guidelines by the South African National Department of Health support the use of a DTG preferred first‐line ARV regimen for all adults living with HIV, including pregnant women. For neonates born to mothers with HIV, a prophylaxis regimen of nevirapine (NVP) and ZDV is routinely started at birth and continued until the maternal viral load result at delivery is available, after which a risk‐stratified approach determines the ARV drug(s) given [15].

In Stage 2 of the PETITE‐DTG trial, eligible low‐risk term neonates with a birth weight ≥2 kg born to virologically suppressed mothers with HIV on DTG‐based antiretroviral therapy (ART) were enrolled. A total of 43 neonates were randomized (1:1) to receive either 5 mg oral dispersible film (DTG‐FILM) (Laurus Labs Ltd., Hyderabad, India) or half of a 10 mg DTG dispersible tablet (DTG‐DT) (Viatris Ltd), on top of ZDV prophylaxis (syrup), for 4 weeks. At the time of the study, the DTG‐Film had received tentative US Food & Drug Administration approval but was not yet commercially available [8]. Forty‐one neonates received at least one dose of DTG, and 39 neonates completed the study. A sub‐set of mothers from both cohorts, sampled for diversity in terms of age and home language, were invited to participate in the qualitative assessment. Here, we report on data from mothers whose neonates received the DTG‐Film as well as health workers involved in the trial.

**FIGURE 1:**
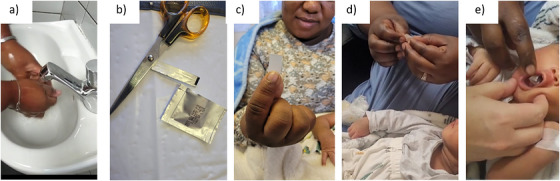
Dolutegravir (DTG)‐Film administration: (a) washing hands; (b) cutting the sachet; (c) mother holding the DTG‐Film; (d) folding the film; (e) administering the DTG‐Film to the neonate.

The DTG‐Film is a thin (1 cm x 2 cm), white, taste‐masked film. During administration, mothers would wash their hands, remove the film from a (cut) foil sachet, fold in half and place it directly on the tongue of the infant by the mother (Figure 1). The first DTG‐Film administration was with guidance from health workers at the trial site. Subsequent administration was conducted at home by the mothers. Infants received 5 mg DTG‐Film every second day until day 14, whereafter dosing was adjusted to include daily DTG administration until day 28. Health workers provided mothers with a diary to support and guide administration to infants (File S1). After day 28, neonates continued with standard‐of‐care HIV prophylaxis as per local guidance [16].

### 2.2 Data Collection

Mothers with neonates enrolled in the PETITE‐DTG trial were invited to participate in three in‐depth and interactive interviews over the course of their neonate's enrolment in the trial. Between September 2023 and November 2024, interviews were conducted at enrolment, focusing on household context and immediate perceptions of the DTG formulation; on day 14, exploring health journeys and follow‐up questions on experiences of DTG administration; and at the end of the study (before 6 weeks of life), exploring health beliefs, treatment preferences (reported elsewhere) and overall treatment experiences.

All interviews were conducted in the language of choice of participants (Afrikaans, English or isiXhosa) by two local, trained, post‐graduate qualitative South African researchers with experience working in health systems in the province. The researchers were an integral part of the PETITE‐DTG study team. They were present when mothers were informed about the study and at the time of on‐site neonatal DTG‐Film administration. They worked closely with the health workers on the study.

Mothers were interviewed at the study site, shortly after the baby's clinical assessment as part of the parent trial. Where possible and where participants agreed, the final interview was conducted at participant homes. Each interview lasted 30–60 min.

Health workers involved in the study were recruited from the trial site and participated in two interviews—at the start and the end of the trial. Interviews were conducted in English, lasted ∼ 30 min and focused on their perceptions, experiences, and preferences of administering HIV treatment and prevention to neonates, including novel DTG formulations.

All interviews were audio recorded, and researchers completed detailed, structured case descriptions in English with supportive verbatim translated quotes [17]. All case descriptions were quality checked by the first author, and regular debrief sessions were held with the field team.

### 2.3 Data Analysis

The study team reviewed all case descriptions during an analysis workshop. We applied a deductive thematic comparative approach based on the treatment acceptability framework proposed by Wademan et al. The framework, developed to understand treatment acceptability among children, comprises the domains of usability, receptivity and integration to gain a comprehensive understanding of acceptability. It considers how factors like palatability, administration and socio‐economic context influence treatment acceptability, uptake and adherence [18]. The analysis team iteratively cross‐checked themes and provided additional verbatim quotes from participants, as needed.

### 2.4 Ethics

The study was approved by the Stellenbosch Health Research Ethics Committee (M22/01/001) as well as the World Health Organization's ethics committee. Mothers with neonates enrolled in the PETITE‐DTG trial were invited to participate in additional in‐depth interviews. Participants were able to refuse participation without compromising their neonate's participation in the parent trial. All mothers signed written informed consent, and consent was reaffirmed during each interview. Participant IDs (PIDs) are used throughout to anonymously report findings. Health workers also signed written informed consent and had the opportunity to opt out.

## 3 Results

Forty‐three neonates were enrolled, and 41 received at least one dose of DTG in the PETITE‐DTG trial. Twenty‐one neonates were randomized to receive the DTG‐Film, with 20 receiving at least one dose. We present findings from 16 virologically suppressed mothers of neonates who received the DTG‐Film for a period of 4 weeks. Fifteen mothers took part in all three interviews, and one mother declined the closing interview but agreed to have her data included for analysis. An in‐home visit was conducted with 5/16 consenting mothers (Table 1).

**TABLE 1:** Characteristics of mother−infant pairs enrolled in the PETITE‐DTG study in the DTG‐Film cohort.

Maternal characteristics		Mothers with neonates in the DTG‐Film cohort (*n* = 16)^a^
Age (*years*)	Median (range)	38 (23−43)
Time since HIV diagnosis *(years)*	Median (range)	8.8 (2−15)
Time on DTG‐based antiretroviral therapy (ART) *(months)*	Median (range)	37.2 (3.0−88.9)
Disclosed HIV status to significant partner (*yes*)		12 (75%)
Number of children	Median (range)	2.9 (1.0−4.0)
Previous experience with using antiretroviral drugs for HIV prevention in their newborns (*yes*)		8 (50%)

**Neonatal characteristics**		**Neonates in the DTG‐Film cohort** ^a^ **(*n* = 16)**
Sex *(male)*		10 (62.5%)
Gestational age at birth *(weeks)*	Median (range)	39.5 (37.3−41.6)
Birth weight *(kilograms)*	Median (range)	3.3 (2.6−4.3)
Age in days of life at randomization	Median (range)	3 (2−4)

^a^ Two mothers had twins, each with one neonate receiving the DTG‐Film and one receiving the DTG tablet. Their reported findings on the DTG‐FILM are included in this analysis.

Abbreviation: DTG, Dolutegravir.

Six health workers were interviewed. All health workers were female and were employed in various roles, including as pharmacist, adherence monitor, medical officer, nurse and project manager. Health workers were aged between 42 and 54 years (median age: 47 years).

### 3.1 Acceptability of the DTG‐Film

We describe (1) the usability of the DTG‐Film and how well the oral film can be integrated into care process for neonates; (2) how receptive mothers (participant code: PID) and health workers (participant code: HW) are to using the novel formulation; and (3) participants’ views on the integration of the film as part of receiving or providing care for neonates.

### 3.2 Usability of the DTG‐Film

To understand the usability of DTG‐Film, we describe the administration practices by mothers and health workers and the overall appeal of the oral film to end‐users.

#### 3.2.1 Usability: Administration

Mothers and health workers described the administration of the DTG‐Film as simple, quick and easy with minimal complications. Prior to administration of the first dose, the clinical staff explained to mothers how to prepare the film for administration (washing and drying hands, opening the sachet with scissors) and how to fold and insert the film into the mouth of the neonate.

Most mothers noted that, even at first use, both the preparation and the actual administration were straightforward. One mother explained:

“I wash my hands … make sure they are dry, and then I just cut the paper [DTG‐Film sachet], fold it [film], and it is quick, so as soon as I put it onto his [infant's] tongue … it dissolves in a few seconds; he enjoys that one” [PID35].

Another mother noted “that thing [film] is easy, you bend it, and put it in [the mouth], he sucks it” [PID 24]. Similarly, a health worker noted that the “DTG‐Film is much easier [than syrups], as long as you have scissors, you just cut [the sachet] and take it [film] and put it in the mouth” [HW01].

Several mothers noted that it took “only a few seconds” to dissolve. Mothers also described how neonates were able to ingest the entire film: “he did not spit it, he did not vomit, he swallowed within two seconds, it dissolves fast, and it's gone in the mouth, it left nothing” [PID23]. One health worker described that she was “so proud of that film … every time I hear a patient gets the film, then … to me it feels like the patient gets the whole dose” [HW04].

Mothers would employ different strategies to ensure that their neonates ingested the entire dose when at home: “What I would do, is, he cries a lot when I bath him, so I would make sure I do the film while he is crying, as soon as I put the film he keeps quiet and starts sucking, and it is 5–10 s, and it would be done” [PID35].

One challenge identified by both mothers and health workers was that, when wet, the film would become sticky, and occasionally attach to either the mother's finger, or the lips or palate of the infants, leaving residue behind. One mother described how the *“[DTG‐Film] was very easy to give and [the neonate] had no problem taking it, except one time she was sleeping, and it got stuck to the palate*” [PID38].

Over the course of the study, health workers gained insight as to how best to administer the film and offered advice, as one mother explained: *“Once it happened that as soon as I administered it [DTG‐Film], I thought it will get stuck there, the doctor said I must breastfeed him so that it can easily get dissolved and go down” [PID39]*. A health worker explained that it takes *“maybe a minute because there is [some film] that is left on the finger and then now I noticed that it sticks, so you have to keep trying for the child to get that bit as well, but not more than a minute” [HW02]*.

Few mothers commented on the dosing adjustment during the first month—once every second day for the first 2 weeks and then daily from day 14 onwards*: “It's going to be easier, because like now I skip a day and forget about it, and when I do it every day, it will become easier, like a routine” [PID34]*. Some of the health workers also expressed concerns:

“Daily [administration], from the beginning would be much easier, as a mom, if I had to remember every second day I would have to have a calendar on the wall where I could mark it off and I would be constantly second guessing when it was administered, I don't think it's really ideal, especially not for a mom with a new baby” [HW01].

However, none of the participants reported major challenges with dosing routines.

Given the novelty of the film, mothers appreciated the guidance from health workers, including written instruction, on administration (see File S1) *and throughout the treatment period, mothers consistently reported that administration was easy: “that first day they helped me just to show me how it is done, they taught me and gave me this [film], there is a paper [pamphlet] that they gave me so that I can read it to know what to do, and they also helped me the first day showing me how it is done” [PID23]*.

#### 3.2.2 Usability: Packaging Appeal

The film was described as appealing by both mothers and health workers, although there were some amenable challenges noted in relation to the packaging that could be improved in future:

“One thing I am finding … the packaging is not great, but I am sure that is something that would be optimized if we are marketing it. You would have to have scissors in order to open it, this one doesn't have a specific …tear or opening … the packaging itself it quite difficult to open” [HW01].

Another health worker summarized: “I mean, who walks around with scissors every day? You know, something that says, ‘tear here’ or ‘cut here’ [would be better]” [HW06]. Mothers, however, did not share the same concerns: “I cut that packet, I noticed that the film is in the middle of the paper, so it is very easy” [PID31]. One mother even noted that, over time, she learned to take scissors with her when not at home: “when I go out somewhere now, I [bring] a small [set of] scissor in my bag just to cut it open, but I don't worry about it, it's just something I have to remember” [PID48].

### 3.3 Receptivity

Receptivity entails the end‐user's willingness to accept an intervention or product [18]. It is shaped by participants’ conceptions of health and, in this case, how the film is expected to prevent HIV transmission. Previous experiences of ARV drug administration, including for use in neonates, also influence receptivity.

#### 3.3.1 Receptivity: Conceptions of Health and DTG‐Film as Medication

Several participants were hesitant when first introduced to the DTG‐Film. None of the participants, including the health workers, had previously come across an oral dispersible film medication. However, after administering the first dose of the DTG‐Film, the general response was positive, with several participants describing the film as their formulation of choice (data published elsewhere). One mother initially described how: “*At first, it did not sit well with me … I have never seen paper being eaten, never*.*”* However, after administering the film at home, the same participant later noted: “*there at home it was easy … you bend that [film] and put it on the tongue*” [PID24].

Families of participants were also unfamiliar with the film, or “the paper” as participants generally referred to it. The mother of a set of twins—one receiving the DTG dispersible tablet and the other receiving the DTG‐Film—noted:

“My parents, with the [DTG] dispersible tablet they were watching me do it and they were like, ‘oh okay’, but with the film they were like, ‘What is this? Why are you doing this? It looks like paper.’ … My mom was worried that the baby might choke, but I showed her that it dissolves [so the baby won't choke]” [PID35].

All mothers noted that they prioritized their neonate's health and that the safety of the film was a primary concern. One mother said: “*The most important thing to me is my child's health, so I want to make sure that she is safe”* [PID27]. Another said: “*I asked questions if it is safe, they said it is safe, so I did not see anything harmful because it is just something that is administered to the tongue*” *[PID39]*. This trust in the novel formulation was grounded in familiarity with and faith in health workers, the opportunity for mothers to ask questions, increased familiarity with the formulation over time, and by observing other mothers in the PETITE‐DTG trial, highlighting the importance of social support/influence:

“I just give them this one (DTG‐Film), because even when we talk there with other mothers [in the waiting room] we see that, there where we sit, we see that this is the one that is good/right for them” [PID32].

When asked how the DTG‐Film functioned, few participants were aware of the technicalities, but all understood the reason for administration and believed that the DTG‐Film would provide protection from HIV for their neonates. They were, however, aware of that protection through the film was not permanent:

“Since I am HIV positive, and I am also breastfeeding, the medication is basically protecting the babies from getting HIV … [it does not protect] … forever, otherwise it would be once and then like never again, like a vaccine, but it has to happen regularly” [PID35].

#### 3.3.2 Receptivity: Prior Experience With ART for Neonates

Of the 16 mothers with infants receiving DTG‐Film, eight had accessed VTP services through routine care during previous pregnancies. Some mothers noted that these experiences with HIV preventive care facilitate acceptability of DTG‐Film:

“I trust it [DTG‐Film] a lot, because there is always a first time with anything, and at the clinic they say you must use it. Since it [ART] has never disappointed me … I trust it” [PID42].

Similarly, another mother noted that:

“I am confident about the medication [my baby] receives because [my older daughter] also received medication and she is still HIV negative so [I] trust that even now it will be like that” [PID29].

Other mothers explained that they were familiar with DTG, as they were, themselves, taking DTG prior to giving birth: “I think they are trying to make it easier, like I think it's the same [medicine], but they are trying to make it easier for the child” [PID31].

### 3.4 Integration

Health workers and mothers described how the DTG‐Film was integrated into their care practices, including how DTG‐Film would be included in existing health systems, and how support would be accessed in home contexts.

#### 3.4.1 Integration: Health System Delivery

Support for administering the DTG‐Film was provided by dedicated study staff at FAMCRU, located at Tygerberg Hospital in Cape Town, alongside frequent study visits. Outside of the trial setting, participants would have accessed health services, including HIV preventative care for their infants, at community‐based primary care facilities (clinics). Participants (mothers and health workers) were asked to reflect on the potential integration of the DTG‐Film in routine health services.

Mothers described the hands‐on and individualized care they received as part of the trial and how this facilitated treatment administration:

“Getting medication at FAMCRU, Tygerberg, it's easy … the doctors at FAMCRU [are] trying to make it [DTG‐Film] easier to give … and [the doctor] said ‘oh mommy show me’, and I … showed her and she said, ‘Wow!’” [PID31]

The availability of regular care was also highlighted by mothers: “at the clinic they tell you to come once a month, skip a month, but here they meet up with you every week” [PID42].

While the care received in the trial setting was described as invaluable to participants, in general, health workers described how the film format of DTG would be especially useful in a public health setting, where other formulations would need manipulation prior to administration:

“With pills, a pharmacist is not going to pop them out and break them for the mom, she would have to do that herself … the film, it's no fuss, no measurement Its accurate, you know exactly what is going in the baby … there is no spill, no fuss, you just give it and carry on with your life … the syrup, it involves measurements which needs focus, you need to trust the mom gives the right volume” [HW01].

Health workers also noted that, while the film would fit with existing programmes, educating clients and training health staff would be key: *“education … on the medicine, with instructions and explanations, it [DTG‐Film] would be easily integrated” [HW04]*.

#### 3.4.2 Integration: Household Support and Discretion

Integrating the DTG‐Film into the households of women living with HIV was facilitated by support received from other household or family members. For instance, one mother explained, *“people in the household are very supportive and feel confident about the medication and oftentimes they would remind [me] about the medication” [PID34]*. Similarly, the husband of another participant was described as “*very supportive and helps to give the medications [to the neonate]” [PID27]*. However, some mothers, specifically those who had not yet disclosed their HIV status to their partners or who were not married, explained that they carried both the burden and responsibility of taking care of their neonates: *“I make the decision alone because I am the mother, we are not married, it's me, I have to make the decision of the child” [PID31]*.

Others noted that the film was discreet and could potentially help to avoid inadvertent disclosure where participants chose not to disclose their HIV status to their partners or other household members. One mother explained how her partner would ask,

“The paper that you keep giving the child, what is it for?’ I say it's things from the doctor; I explained to him … it is ‘medicine’ and he let it go; he does not know about [my] HIV status” [PID24].

While another noted:

“With people that one has not disclosed to, then the DTG‐Film would be best … [I] can easily administer medication without fear of being seen” [PID32].

Generally, mothers described how support from household and family members facilitated treatment integration into the household. Simultaneously, the discreetness of the film enabled those who did not have support to integrate the film in their households while limiting the risk of inadvertent disclosure.

## 4 Discussion

Overall, the DTG‐Film was described as highly acceptable to virologically suppressed mothers of neonates enrolled in the PETITE‐DTG trial and health workers familiar with the formulation. Mothers and health workers found the film to be quick and easy to administer, fast to dissolve and simple to handle. After some initial hesitation due to unfamiliarity with the film, mothers gave positive feedback. Mothers and health workers were receptive of the film and described it as discreet, and easy to integrate, especially with support from household members or significant others. Participants also noted that they had some concerns with relation to the film “getting wet” and sticking either to the mother's fingers, or the infant's lips. However, participants were confident that the infant received the entire dose. Participants had limited concerns on the packaging that had to be opened with scissors.

A previous review of oral films used across contexts and formulations, for diverse health conditions and different end‐users, found that taste, texture, mouthfeel and dissolution times are key in determining acceptability [10]. Similarly, Scarpa and colleagues conducted a study with healthy young adults to determine key acceptability criteria of placebo oral dispersible films and found that perceived stickiness in the mouth and disintegration time were key determinants for end‐users [19]. However, these features are often difficult or not appropriate for assessment in younger children. Important features driving the acceptability of oral films include ease of administration, the reduced risk of choking and fast disintegration directly in the mouth [20].

Few studies have explored mothers and/or caregiver and health worker perceptions on the acceptability of oral films in infants or neonates, although those that do consistently report positive responses towards the acceptability of these formulations. For instance, in one study, placebo oral dispersible films were administered to infants (>6 months) and children (<5 years) with acute illness or long‐term stable condition in a hospital setting in the United Kingdom. Findings showed that the majority of children and caregivers, including mothers of infants, responded positively towards the administration of oral dispersible films [21]. Similarly, Klingmann and colleagues found glucose oral dispersible film to be non‐inferior to glucose syrup in terms of acceptability when administered in infants and neonates (2 days up to 12 months). The film also scored better when swallowability and palatability were assessed [12]. Our findings are also reflected in the preferences study conducted by Wexler and colleagues in Kenya. They identified key attributes impacting the preferences of oral films among health workers and caregivers for paediatric use. This includes rapid dissolution, small size, and no special storage requirements, and packaging that is easy to open. In addition, and not specifically mentioned in our study, end‐users commented on taste, single film doses and waterproof packaging [22]. Overall, our findings provide additional support for the use of oral dispersible film for administration in neonates, with ARV administration reported as feasible and acceptable by both mothers and health workers. Development of a 2.5 mg DTG‐Film administered daily would simplify administration for mothers in the first 2 weeks of life. More user‐friendly packaging, that is easy to tear, training of health workers and peer support for mothers would all assist with the swift adoption of this novel formulation in future. Administration instructions for the DTG‐Film should include folding the film to fit in the neonate's mouth, how to avoid getting the film wet by placing the film directly onto the tongue, encouraging feeding after administering the film and including reassuring language on dosing accuracy, despite the unfamiliar formulation modality.

As a strength, the study employed a longitudinal approach, and interviews were conducted in the home language of participants, which meant that researchers were able to build rapport with participants. The positionality of the data collection team also strengthened the findings. Researchers were female, of the same age groups as the mother participants, local, familiar with the hospital setting and fluent (first language) in the same language as the mothers. The researchers spent extensive time at the research site, ensuring that mothers and health workers were familiar and comfortable with them. Central to their training, and our approach to data collection, was building relationships centred around empathy and care. Where possible, researchers conducted home visits, furthering contextual understanding of participants’ experiences. The research team had regular debrief sessions and applied an iterative approach to analysis, often reflecting on the relationships they had with the mothers. Another strength is that this is the first time an ARV oral film has been tested in neonates, and to date, the film has only been tested in healthy adults in a bioequivalence study. Through this in‐depth analysis, we are able to show how the novel formulation is received by both health workers and mothers administering the DTG‐Film.

As a limitation, this analysis was conducted at a single site in South Africa. However, this study provides initial but important insight into the acceptability of novel ARV oral films in neonates, as perceived by mothers and health workers. The assessment was conducted in a trial context where mothers received additional support, and mothers who receive care for neonates in routine settings might experience more challenges to administration, access and support. Additionally, health workers interviewed were those who were working on the trial and who were familiar with the DTG‐Film. However, all health worker participants had extensive experience working in public health and were able to compare the DTG‐Film with standard‐of‐care syrups available in routine settings.

## 5 Conclusions

The DTG‐Film provides neonates with a potent and safe ARV option that is highly acceptable for both mothers and health workers. Uptake and adherence of neonatal ARVs are critical in the postpartum period, when mothers must balance the care of their newborn with their own health needs. The DTG‐Film formulation offers a simple, easy‐to‐administer and acceptable alternative to mothers. Future research should focus on developing formulations suitable for once‐daily dosing, which could further simplify administration in the first 2 weeks after birth, potentially improving adherence and enhancing protection against HIV acquisition.

## Author Contributions

All authors have read and approved the final manuscript. LV: conceptualization, methodology, investigation, validation, formal analysis, supervision, writing – original draft. CP: investigation, validation, formal analysis, writing – review and editing. VL: investigation, validation, formal analysis, writing – review and editing. SdT: writing – review and editing. MG: writing – review and editing. LG: writing – review and editing. ACH: funding acquisition, writing – review and editing. AJG–P: funding acquisition, writing – review and editing. TRC: funding acquisition, writing – review and editing. AB: supervision, funding acquisition, writing – review and editing.

## Conflicts of Interest

The authors have no conflicts of interest to declare.

## Supporting information




**Supporting File S1**: Study medication diary card for the PETITE DTG trial.

## Data Availability

The data that support the findings of this study are available on request from the corresponding author. The data are not publicly available due to privacy or ethical restrictions.
