# Supplementary material for: Acceptability of a Dolutegravir Oral Dispersible Film in Term Neonates Born to Mothers Living With HIV
Source: J Int AIDS Soc. 2026 Jun 19;29(Suppl 1):e70104. doi: 10.1002/jia2.70104 (PMC13281408; doi:10.1002/jia2.70104)
Supplement: Supplementary file 1 — Supporting File S1: Study medication diary card for the PETITE DTG trial. [file JIA2-29-e70104-s001.docx]

**Supplementary File 1: Study Medication Diary Card for the PETITE DTG Trial**

**PETITE-DTG Study Medication Diary Card and Administration Pamphlet for Caregivers - English**

**V1.0, dated 12 Sep 2023**


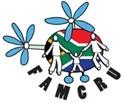


**PETITE-DTG Study**

**Study Medication Diary Card**

| **STUDY NUMBER (PID)** |  |
| --- | --- |
| **DATE ENROLLED** |  |

**FAMCRU**

**Please contact us if there are any problems:**

| **Unit secretary:** |  |
| --- | --- |
| **Study doctor:** |  |
| **Pharmacy:** |  |
| **Cellphone:** |  |

**Thank you!**

**How to Complete this Diary:**

- Please complete this form with a **pen**.
- Mark with “**✓**” and write the time when you give the medicine in the morning.
- Give medication every 2^nd^ day for the first two weeks and then every day for the following two weeks.
- If baby vomits, please make a note of the date and time and inform the study team.

|  | **DATE** | **Dose number** | **Time** | **Given** | **Not given** | **Reason if not given** | **Any vomiting within 30 minutes of giving medicine?** |
| --- | --- | --- | --- | --- | --- | --- | --- |
| Thurs | **03 Oct 2024** | 1 |  |  |  |  |  |
| Fri | **04 Oct 2024** | **skip** |  |  |  |  |  |
| Sat | **05 Oct 2024** | 2 |  |  |  |  |  |
| Sun | **06 Oct 2024** | **skip** |  |  |  |  |  |
| Mon | **07 Oct 2024** | 3 |  |  |  |  |  |
| Tues | **08 Oct 2024** | **skip** |  |  |  |  |  |
| Wed | **09 Oct 2024** | 4 |  |  |  |  |  |
| Thurs | **10 Oct 2024** | **skip** |  |  |  |  |  |
| Fri | **11 Oct 2024** | 5 |  |  |  |  |  |
| Sat | **12 Oct 2024** | **skip** |  |  |  |  |  |
| Sun | **13 Oct 2024** | 6 |  |  |  |  |  |
| Mon | **14 Oct 2024** | **skip** |  |  |  |  |  |
| Tues | **15 Oct 2024** | 7 |  |  |  |  |  |
| Wed | **16 Oct 2024** | 8 |  |  |  |  |  |
| Thurs | **17 Oct 2024** | 9 |  |  |  |  |  |
| Fri | **18 Oct 2024** | 10 |  |  |  |  |  |
| Sat | **19 Oct 2024** | 11 |  |  |  |  |  |
| Sun | **20 Oct 2024** | 12 |  |  |  |  |  |
| Mon | **21 Oct 2024** | 13 |  |  |  |  |  |
| Tues | **22 Oct 2024** | 14 |  |  |  |  |  |
| Wed | **23 Oct 2024** | 15 |  |  |  |  |  |
| Thurs | **24 Oct 2024** | 16 |  |  |  |  |  |
| Fri | **25 Oct 2024** | 17 |  |  |  |  |  |
| Sat | **26 Oct 2024** | 18 |  |  |  |  |  |
| Sun | **27 Oct 2024** | 19 |  |  |  |  |  |
| Mon | **28 Oct 2024** | 20 |  |  |  |  |  |
| Tues | **29 Oct 2024** | 21 |  |  |  |  |  |
|  |  |  |  |  |  |  |  |

# Instructions for Administration of the DTG-Film

**How to give the DTG-Film to your baby:**

1. Ensure your hands are clean and **dry** before handling the DTG-film so the film does not stick to your fingers
2. Open the pouch and take out the film
3. Immediately place the film carefully on top of your baby’s tongue
4. The film will stick to the tongue and begin to disintegrate or dissolve
5. Your baby can close their mouth and swallow normally

# ‘Top Tips’ for Administration of the DTG-Film

1. Fold the film, either in half or quarters, to fit in the baby’s mouth.
2. Place the film directly onto the tongue to avoid getting the film wet.
3. Feeding the baby after giving the film may be helpful
4. Remember, the film **is** medication and your baby is getting the entire dose when you put the film directly on their tongue.
5. Ask for help if you have any questions – we are here to help!
